# Youth HIV services in the context of COVID-19 pandemic in Sofala Province, Mozambique: Virginia Casigliani

**DOI:** 10.1093/eurpub/ckac130.037

**Published:** 2022-10-25

**Authors:** V Casigliani, N Ronzoni, A Merolle, F Chenene, V Cinturao, G Putoto

**Affiliations:** 1 Translational Research and New Technologies in Medicine and Surgery, University of Pisa, Pisa, Italy; 2 Medici con l'Africa CUAMM, Padua, Italy

## Abstract

**Background:**

After the first COVID-19 case in Mozambique, the government established a state of emergency in period April - September 2020. To reduce exposure for people living with HIV (PLWHIV), the Ministry of Health modified service delivery guidance, interrupting community activities, and revising patient flow within health facilities. The study aimed to measure the impact of the COVID-19 pandemic on HIV testing and treatment services in Sofala Province, Mozambique.

**Methods:**

The study analysed the activities in 9 youth HIV services called SAAJs (Serviço amigo do adolescente e jovem) supported by Doctors with Africa CUAMM in 2020 and 2021. The following data were gathered and analysed: number of counselling sessions, number of HIV tests performed, number of people who tested positive and therefore started the antiretroviral treatment (ART), number of PLHIV on ART. Data were disaggregated by sex and age.

**Results:**

In 2020 and 2021 85466 and 141844 counselling sessions were performed, respectively. A decrease of 41% was observed in the second trimester of 2020 compared with the previous one. The number of counselling sessions came back at pre-pandemic levels in the 2nd trimester of 2021. People aged 20-24 accessed more in 2020, while those aged 15-19 in 2021. In 2020 people tested for HIV were 22753, while the number was twice in 2021: the increase was higher among males(p<.05). In 2020 females were more likely to be tested, while in 2021 it was the opposite(p<.05). The positivity rate was 2.5% and 1.5%, respectively; in both years males were more likely to be tested positive(p<.05). In 2020 86.1% of people tested positive started the ART, in 2021 98%. Males were more at risk of not starting the ART(p<.05). The number of PLWHIV on ART did not decrease over time.

**Conclusions:**

ART provision was generally maintained during the COVID-19 pandemic, while other services were heavily impacted. The difference observed among sex was significant and may inform future interventions.

**Key messages:**

- The pandemic impacted severely HIV services in 2020, especially counselling and testing activities.
- It is relevant to assess how the population responded to services’ restoration.

